# Advances in Immune Monitoring Approaches for Sepsis-Induced Immunosuppression

**DOI:** 10.3389/fimmu.2022.891024

**Published:** 2022-05-10

**Authors:** Ren-Qi Yao, Chao Ren, Li-Yu Zheng, Zhao-Fan Xia, Yong-Ming Yao

**Affiliations:** ^1^ Translational Medicine Research Center, Medical Innovation Research Division and Fourth Medical Center of the Chinese People's Liberation Army (PLA) General Hospital, Beijing, China; ^2^ Department of Burn Surgery, The First Affiliated Hospital of Naval Medical University, Shanghai, China; ^3^ Department of Pulmonary and Critical Care Medicine, Beijing Chaoyang Hospital, Capital Medical University, Beijing, China

**Keywords:** sepsis, immunosuppression, biomarker, immune monitoring, innate immunity, adaptive immunity

## Abstract

Sepsis represents a life-threatening organ dysfunction due to an aberrant host response. Of note is that majority of patients have experienced a severe immune depression during and after sepsis, which is significantly correlated with the occurrence of nosocomial infection and higher risk of in-hospital death. Nevertheless, the clinical sign of sepsis-induced immune paralysis remains highly indetectable and ambiguous. Given that, specific yet robust biomarkers for monitoring the immune functional status of septic patients are of prominent significance in clinical practice. In turn, the stratification of a subgroup of septic patients with an immunosuppressive state will greatly contribute to the implementation of personalized adjuvant immunotherapy. In this review, we comprehensively summarize the mechanism of sepsis-associated immunosuppression at the cellular level and highlight the recent advances in immune monitoring approaches targeting the functional status of both innate and adaptive immune responses.

## Introduction

Sepsis is characterized as a life-threatening organ dysfunction due to dysregulated host response to infection based on the definition of the Third International Consensus Definitions for Sepsis and Septic Shock (Sepsis 3.0) (1). Sepsis represents a global healthcare problem imposing enormous economic and societal burdens since it is the most common cause of in-hospital and intensive care unit (ICU) mortality (2). The complex host immune response during sepsis involves the concomitant presence of both pro-inflammatory and anti-inflammatory responses but manifesting a disturbed homeostasis, in association with excessive tissue damage and even organ failure (3). Although the onset and progression of sepsis is substantially heterogeneous across disparate populations, the occurrence of severe immunosuppression is consistently observed in most septic patents, which appears to be significantly correlated with deteriorative clinical outcomes (4, 5). However, the exact mechanism underlying sepsis-induced immunodeficiency has not been established for decades.

Robust yet feasible immune monitoring methods are currently lacking in clinical practice, rendering us unable to timely recognize the immunosuppressive status of septic patients (6, 7). Thus, specific and sensitive biomarkers are urgently required to monitor the immune status of patients with sepsis and septic shock, which is also the prerequisite for the development of novel tailored immunotherapies. In light of the evidence provided by translational and clinical studies, the current literature review summarizes the characteristics of sepsis-induced immune dysregulation at the cellular level and focuses on the recent advances concerning the immune monitoring measures for septic patients, which intend to help clinicians revisit sepsis-associated immunosuppression in depth (8). Since the most comprehensive review by Venet et al. was published almost 10 years ago, this study might represent an updated version of such review, addressing identical topics (9).

## Characteristics of Sepsis-Induced Immunosuppression

**Figure 1:**
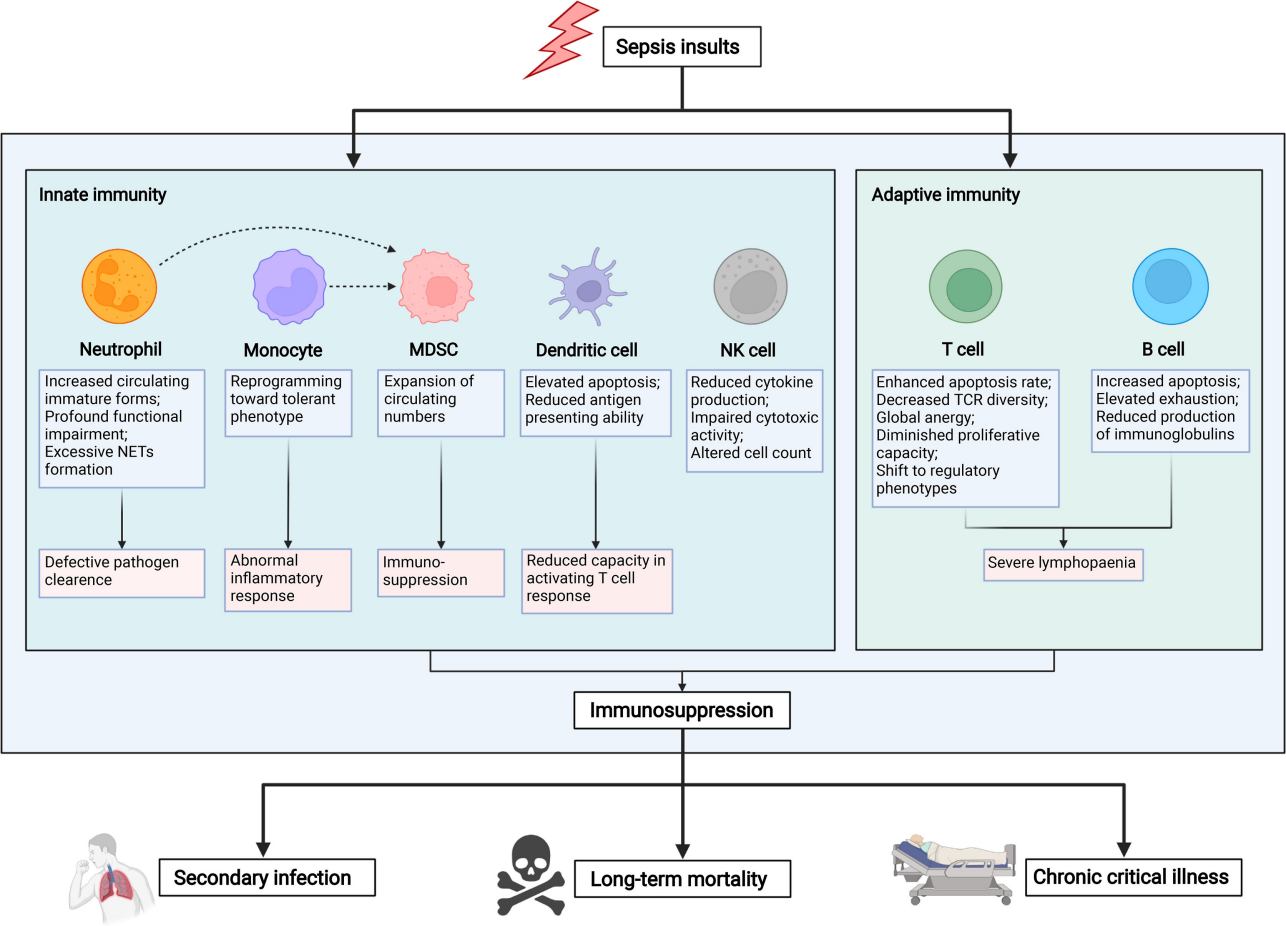
Mechanisms and hallmarks of sepsis-induced immunosuppression. The innate and adaptive immune responses are significantly altered upon septic insults. With regard to innate immunity, sepsis induction results in a substantially increased apoptotic rate across various innate immune cell subsets, including neutrophils, monocytes, dendritic cells, and natural killer cells. Nevertheless, monocytic and granulocytic myeloid-derived suppressor cells have consistently undergone a profound augmentation, as evidenced by elevated circulating numbers in septic patients. As for adaptive immune response, both T and B lymphocytes are presented with significant apoptosis and functional anergy. Meanwhile, a phenotypical shift from effector subtypes to regulatory subtypes can be commonly observed in patients with sepsis. In turn, tremendous lymphopenia largely contributes to the development of sepsis-induced immunosuppression, thereby leading to increased risk of nosocomial infection, chronic critical illness, and even long-term mortality. Graph was created with BioRender.com. NETs, neutrophil extracellular traps; MDSCs, myeloid-derived suppressor cells; NK cells, natural killer cells; TCR, T cell receptor.

Immune suppression in sepsis is noted with increased susceptibility of patients to secondary and nosocomial infections, thereby leading to elevated readmission rates and deteriorative long-term mortality (10, 11). Diverse yet intricate mechanisms have been demonstrated to be involved in the development of sepsis-associated immune dysregulation, including cellular apoptosis, autophagy, endotoxin tolerance, metabolic reprograming, and epigenetic regulation (5, 12, 13). Meanwhile, it should be noted that the interplay between these mechanisms has also been characterized in the immune dysfunction after sepsis (5). One major example represents endotoxin tolerance, manifesting as diminished proinflammatory cytokine production of various myeloid cells in response to a re-challenge of endotoxin (lipopolysaccharide, LPS) or other stimuli (14). Multiple studies have confirmed that both metabolic reprograming and epigenetic regulation are critically involved in the establishment of endotoxin tolerance as evidenced by substantial alterations in the transcription of genes encoding deacetylase enzymes and hypoxia-inducible factors (15–17). A reinforced host response to secondary stimuli in innate immune cells, namely “trained immunity”, has been likewise demonstrated to participate in the progression of sepsis and excessive tissue damage (18, 19). Other than histone modifications, DNA methylation and shift of metabolic profile were reportedly responsible for the process of trained immunity, which could be transmitted to daughter cells (19, 20). Since innate immune memory exerts a potent effect against systemic infection, the maladaptation of these mechanisms greatly contributes to post-sepsis immunosuppression. More importantly, immunosuppression can be largely attributed to the dysfunction of various immune cell types since septic insults substantially affect both the innate and the adaptive immune systems (**Figure 1**).

## Innate Immunity

### Neutrophils

As the first-line defense cells in response to pathogens, neutrophils play a pivotal role in innate immunity. Under septic exposure, increased granulocytes can result in excessive release of immature forms of neutrophils in circulation, the existence of which has been demonstrated to impair T cell activation with deteriorative clinical outcomes (21, 22). With regards the relevant mechanism, neutrophils isolated from septic patients exert profound functional alterations, including diminished chemotaxis, impaired transmigration, and decreased oxidative burst, thereby leading to impaired functional capacity of pathogenic clearance (23, 24). Meanwhile, the formation of neutrophil extracellular traps (NETs) and delayed neutrophil apoptosis were closely related to prolonged endothelial and tissue damage and, ultimately, organ dysfunction (25–28). Of note is that the increased number of immature or immunocompromised neutrophils reportedly accelerated the progression and correlated with a high risk of death among patients with sepsis and septic shock (29).

### Monocytes and Macrophages

Monocytes and macrophages are key players in both innate and adaptive immune response, with high heterogeneity and potent immunogenicity. The reduced responsiveness of monocytes could be identified after septic induction, as supported by the decreased production of pro-inflammatory cytokines as well as the enhanced capacity in releasing anti-inflammatory mediators upon endotoxin challenge, which closely resembles endotoxin tolerance (15, 30). The tolerant monocytes from septic patients showed a compromised ability to eliminate internalized pathogens, which could be largely attributed to substantial impairment in the phagocytic capacity of monocytes (17, 31). Additionally, the patrolling, chemotaxis, and antigen-presenting capacities were significantly altered for endotoxin-tolerant monocytes derived from septic patients (32, 33). Notably, macrophages carry out a reprogramming toward an immunosuppressive phenotype in the development of sepsis, which potentiate sepsis-induced immune depression (4, 34). Nevertheless, excessive activation of macrophages can lead to a hyperinflammatory state in sepsis, namely, macrophage activation syndrome (MAS), in association with organ dysfunctions and early death (35, 36). Given that, the selection of molecules targeting macrophage polarization and functions is of great clinical significance in seeking novel measures for diagnosis and the treatment of septic complications.

### Myeloid-Derived Suppressor Cells

Myeloid-derived suppressor cells (MDSCs) are heterogeneous subsets of immature myeloid cells exerting immunosuppressive functions on both innate and adaptive immunity, and two major subpopulations have been identified in terms of granulocytic MDSCs (G-MDSCs) and monocytic MDSCs (M-MDSCs) (34, 37, 38). MDSCs are extensively studied in various malignancies, whereas their potential roles remain poorly understood in the pathogenesis of sepsis. Notably, massive expansion of circulating MDSCs could be frequently observed among septic patients, and they were found to be correlated with chronic immune depression, thereby leading to the development of nosocomial infections after the onset of sepsis (39, 40). Nevertheless, specific markers in defining human MDSCs currently remain lacking, which might restrict us from exploring the role and significance of such cell type in depth (41).

### Dendritic Cells

Dendritic cells (DCs) are known to be the most potent antigen-presenting cells (APCs), and they serve as the bridge linking innate immunity with adaptive immune response *via* its unique capacity in priming naïve T lymphocytes (42). Evidently reduced counts of DCs owing to sepsis-induced apoptosis could be seen in both peripheral blood and spleen from septic patients and those subjected to severe trauma or burns, which were reportedly associated with an increased risk of nosocomial infections and death (43, 44). Moreover, the plasmacytoid and myeloid DC numbers were simultaneously diminished in the setting of sepsis (44, 45). Additionally, functional loss and inability of DCs were frequently reported in sepsis, as evidenced by reduced cytokine secretion and blunted antigen-dependent response, together with their decreased capacity in activating T cell response and propensity to induce T cell exhaustion (46–49). Therefore, altered DC function is responsible for the incapacity of the host against infection, thereby resulting in increased mortality rate among septic patients (50). Given its pivotal role in orchestrating host immune response, the cellular loss and impaired function of DCs significantly contribute to the development of sepsis-induced immunosuppression.

### Natural Killer Cells

As one of innate-type lymphocytes, natural killer (NK) cells are critically involved in host immune response *via* the production of various cytokines and chemokines, which reportedly play an essential role in disparate phases of sepsis progression (51, 52). Upon the onset of sepsis, cytokine production of NK cells was greatly diminished due to endotoxin tolerance, especially for interferon (IFN)-γ (53). Besides this, NK cell cytotoxic activity is substantially impaired in septic insults, implying that immunosuppressive NK cell response might precede the development of sepsis (54–56). Consistent with these findings, alterations of NK cell counts have been demonstrated to correlate with increased early mortality in septic patients (57).

## Adaptive Immunity

### T Lymphocytes

A substantial decline in lymphocyte (specifically CD4^+^ T lymphocytes) counts is well characterized in sepsis (58, 59). Strikingly, sepsis-associated lymphopenia is much more prominent in patients who die from sepsis compared to septic survivors (60). CD4^+^ T lymphocytes that survive from sepsis-induced apoptosis reveal anergic profiles, including diminished proliferative capacity, reduced ability to produce effector cytokines, and upregulated expression of various co-inhibitory receptors that inhibit T cell response (61–63). Moreover, unresponsiveness of T cell receptor (TCR) clonal repertoire and decreased TCR Vβ diversity have been observed in septic patients, and they are positively correlated with an increased risk of nosocomial infections and mortality (64). Following the occurrence of sepsis, CD4^+^ T lymphocytes reportedly have undergone a phenotypical shift from helper T cell (Th) 1 to Th2 subset that possesses a decreased secretion of IL-2 and IFN-γ and impaired proliferative capacity (5, 34, 65). Several studies have confirmed that both Th1 and Th2 differentiations are significantly inhibited during and after sepsis subsides, as supported by the decreased production of Th1- and Th2-related cytokines and the reduced activity of transcriptional factors modulating Th1 as well as Th2 responses (34, 66). Similarly, naïve and memory CD8^+^ T cells are manifested to display profound exhaustion during and after sepsis, with ineffectiveness in mounting a response to emerging antigens (67, 68). In addition to numerical loss, major defects in T cell phenotype as well as functional status give rise to the post-sepsis immunosuppression and deterioration of host immune response. The impact of sepsis on non-conventional T cell subsets has also been well established, especially for the Th17 subset. Th17-associated cytokine secretion is markedly reduced upon sepsis, which reportedly has an adverse effect on long-term mortality (69). As a potent regulator of adaptive immunity, regulatory T cells (Tregs) participate in suppressing the proliferation of other effector T cell (Teff) subsets and mediating the phenotypical shift of Th in the development of sepsis *via* the production of various types of inhibitory cytokines (52, 70). The expansion of the Treg population can be observed after the occurrence of sepsis, which is more prominent in septic patients who died during hospitalization (71, 72). Meanwhile, counts of Tregs are noted to negatively correlate with Th counts, suggesting that Tregs remain resistant to sepsis-induced apoptosis (70). The interrelationship between Tregs and Th17 cells has long been proposed, in which they function antagonistically during the course of sepsis but share similarities regarding different directions. Besides this, sepsis has a substantial impact on various types of innate-like T lymphocytes, including gamma delta T cells (γδ T cells), natural killer T (NKT) cells, and mucosal-associated invariant T (MAIT) cells (73). Similar to other subsets of T lymphocytes, circulating counts of γδ T, NKT, and MAIT cells showed a significant decline following the occurrence of sepsis, the extent of which was associated with an increased risk of infections (74).

### B Lymphocytes

B lymphocytes exert varied functions in the development of septic complications, which are capable of modulating innate immunity and cytokine induction, and function as APCs (75, 76). An evident decline in B cell counts has likewise been reported in human sepsis, secondary to increased apoptosis and T lymphocyte deficit (77). Of note is that the numerical loss of B cells is inconsistent across subpopulations, with a greater apoptotic rate in activated memory B cells than in other subsets of B cells, in association with a long-term risk of recurrent infection among septic survivors (78). Meanwhile, sepsis is associated with a marked decrease in naive B cell number and elevated B cell exhaustion in peripheral blood derived from septic patients, implying a major inability of B lymphocyte to mount an effective adaptive immune response (79, 80). The profound alterations of T cell have been indicated to impair the T lymphocyte-dependent peripheral maturation of B cells, in association with incompetent B cell functions (3, 78). As the major effectors of B cells, immunoglobulins have shown beneficial effects in alleviating endothelial injury as well as facilitating platelet count restoration among septic patients, thereby improving their hemorrhagic tendency (81). Since the impact of intravenous immunoglobulins on the host immune system is of discrepancy, its clinical efficacy in treating sepsis and septic shock remains controversial.

## Monitoring the Alterations of the Innate Immune System

Innate immune cells play an indispensable role in the first-line defense following an infection *via* mediating the pathogenic clearance and regulating the adaptive immune response. Since sepsis induction inevitably leads to the dysregulation of innate immune system, alterations in functional status and imbalance across subpopulations represent pivotal indicators for the monitoring of innate immune response in septic patients (30). Correspondingly, numerous clinical studies have documented encouraging results, and potential indicators are found to be useful for the early diagnosis of sepsis and prediction of deteriorative outcomes (**Table 1**). One major hallmark of an innate immune cell after sepsis represents endotoxin tolerance, which can be commonly observed in monocytes and macrophages. To be specific, monocytes isolated from septic patients manifest a significantly diminished capacity in generating inflammatory cytokines in response to LPS stimulation, including IL-1, IL-6, IL-12, and tumor necrosis factor-α (TNF-α), in association with the development of hospital-acquired infections and deleterious clinical outcomes (15).

**Table 1:** Immune monitoring indicators in human sepsis.

	Immune cell types	Category	Monitoring indicators	References
Innate immunity	Neutrophils	Functional defects	Decreased bactericidal capacity	(82, 83)
			Impaired chemotactic function	(84, 85)
			Decreased spontaneous motility	(86, 87)
			Excessive NET formation	(88)
			Upregulated expressions of CD64, PD-L1, sTREM-1, and HBP	(82, 89–93)
		Alterations in subsets	Increased representation of immature circulating neutrophils	(22–24, 94)
			Increased representation of OLFM4^+^ neutrophils	(95, 96)
	Monocytes/macrophages	Functional defects	Diminished expression of mHLA-DR	(57, 97, 98)
			Upregulation of PD-L1	(99)
			Elevated MDW level	(100–103)
			Decreased production of TNF-α and IL-12	(104)
			Increased secretory level of IL-10	(105)
			Elevated circulating level of presepsin	(106, 107)
			Elevated plasma level of ferritin, IL-6, IL-18, and sCD163	(35)
			Impaired phagocytic capacity	(108)
		Alterations in subsets	Increased proportion of circulating CD14^+^HLA-DR^low^ monocytes	(109, 110)
			Increased percentage of CD14^-^CD16^+^ patrolling monocytes	(33, 111)
	MDSCs	Functional defects	Increased levels of S100A12, S100A8/A9, ARG1, and LOX-1	(112, 113)
		Alterations in subsets	Expansion of G-MDSCs and M-MDSCs	(39, 114)
	Dendritic cells	Functional defects	Down-regulated expression of HLA-DR	(115)
			Enhanced production of IL-10	(49)
			Elevated level of Blimp1 in circulating DCs	(116)
		Alterations in subsets	Reduction of pDCs and mDCs counts	(44, 45)
			Increased representation of BTLA^+^ mDCs	(117)
	NK cells	Functional defects	Inhibitory secretion of IFN-γ and TNF-α	(56, 118, 119)
			Impaired killing capacity	(118)
			Dampened expression of NCRs and NKG2 receptors	(118, 120)
		Alterations in subsets	Reductions in both CD56^hi^ and CD56^low^ NK cells	(121, 122)
			Increased percentage of PD-L1^+^ NK cells	(123)
Adaptive immunity	Total lymphocytes		Persistently low counts of lymphocytes	(60, 124)
	T lymphocytes	Functional defects	Decreased TCR diversity	(29, 64, 125)
			Impaired proliferative capacity	(126)
			Upregulation of exhaustion markers, including PD-1, 2B4, and BTLA	(62, 63, 127–129)
			Inhibitory capacity in releasing cytokines, including IL-2, IL-6, IFN-γ, and TNF-α	(34, 130)
		Alterations in subsets	Reduced ratio of CD4^+^/CD8^+^ T cells	(131–134)
			Imbalanced ratio of Th1/Th2	(135)
			Decreased representation of Th1, Th2, and Th17 subtypes	(105, 136)
			Increased percentage of Tregs	(71, 72, 136, 137)
			Reversed ratio of Th17/Treg	(138, 139)
			Numerical loss of MAIT and γδ T cells	(74)
	B lymphocytes	Functional defects	Increased B cell exhaustion	(140, 141)
			Abnormal level of IgG	(142, 143)
			Decreased level of IgM	(80)
		Alterations in subsets	Upregulated expression of CD80 and CD95 with downregulation of CD23 on B cells	(144)
			Decreases in percentages of circulating plasmablasts and memory B cells	(78, 145)
			Increased percentage of Bregs	(146)

NETs, neutrophil extracellular traps; PD-L1, programmed cell death 1 ligand-1; TREM-1, triggering receptor expressed on myeloid cell-1; HBP, heparin-binding protein; OLFM4, olfactomedin-4; HLA-DR, human leukocyte antigen DR; MDW, monocyte distribution width; TNF-α, tumor necrosis factor-α; IL-12, interleukin 12; MDSCs, myeloid-derived suppressor cells; ARG1, arginase 1; LOX-1, lectin-type oxidized LDL receptor 1; G-MDSCs, granulocytic myeloid-derived suppressor cells; M-MDSCs, monocytic myeloid-derived suppressor cells; pDCs, plasmacytoid dendritic cells; mDCs, myeloid dendritic cells; BTLA, B and T lymphocyte attenuator; NK cells, natural killer cells; IFN-γ, interferon-γ; NCR, natural cytotoxicity receptors; NKG, NK group 2 member; TCR, T cell receptor; PD-1, programmed death-1; Th cells, helper T cells; Tregs, regulatory T cells; MAIT cells, mucosal-associated invariant T cells; γδ T cells, gamma delta T cells; Bregs, regulatory B cells.

### Monitoring the Function and Proportion of Neutrophils

The chemotaxis, phagocytosis, motility, and bactericidal functions are major indicators of the immune responses of neutrophils. The chemiluminescence intensity reflecting the bactericidal capacity of neutrophils showed a significant decrease in septic patients, which was correlated with the severity and poor prognosis of sepsis (24, 82, 83). Neutrophil chemotaxis activity, measured by transmigration assay, was found to independently correlate with 28-day mortality in critical illness with sepsis (84). A recently published study proposed an analyzing platform for the dynamic assessment of neutrophil chemotaxis, in which they revealed an obviously altered chemotactic function of neutrophils in patients complicated with severe infection by applying quantitative indicators (85). Daniel’s team likewise reported that spontaneous motility assessment of neutrophils showed great prospects in accurately yet feasibly identifying populations at a higher risk of developing sepsis (86, 87). Considering the pivotal role of NETs in host defense against infection, Abrams et al. established a novel assay in measuring NET formation based on a prospective cohort study in 341 ICU patients. By incubating plasma with isolated neutrophils *in vitro*, they found a significantly potent NET formation in septic patients, the degree of which could predict the development of disseminated intravascular coagulation and 28-day mortality (88).

CD64 and human triggering receptor expressed on myeloid cells-1 (TREM-1) reflecting the function of neutrophils can serve as potential biomarkers for sepsis. Septic patients were noted with a higher CD64 expression on neutrophils than did non-septic patients at ICU admission. A cutoff of 230 median fluorescence intensity in CD64 expression showed good performance in identifying sepsis (89). It was also reported that the levels of secretory TREM-1 (sTREM-1) in both serum and urine showed higher sensitivity than white blood cell count, C-reactive protein, and procalcitonin in the early recognition of sepsis (82). Heparin-binding protein (HBP) represents another inflammatory mediator released upon neutrophil activation, in association with increased vascular permeability. High plasma levels of HBP could be applied as a marker for the early diagnosis and prognosis of sepsis and septic shock (90–92). In addition, the upregulation of programmed cell death 1 ligand 1 (PD-L1) on neutrophils was identified as a predictor for the prognosis of severe sepsis with persistent immunosuppression. Neutrophils expressed by PD-L1 might comprise a subset that exerted potent inhibitory effects on lymphocytes (93). The number of immature circulating neutrophils, as characterized by CD10^low^CD16^low^ cells, significantly expanded within the first week after the onset septic shock, which was confirmed to correlate with a high risk of immunosuppression of T lymphocytes and worsening among patients with septic shock (22–24). By using cytometry with time-of-flight high-dimensional technology, a recent study identified two novel immature neutrophils subsets—CD10^-^CD64^+^PD-L1^+^ and CD10^-^CD64^+^CD16^low/-^CD123^+^—for the early recognition of a septic complication (94). Moreover, consecutive studies revealed that the increased representation of olfactomedin-4 (OLFM4)^+^ neutrophils was significantly associated with a high risk of short-term mortality in sepsis and septic shock patients (95, 96).

### Monitoring the Function and Proportion of Monocytes as Well as Macrophages

The expression of human leukocyte antigen DR (HLA-DR) is commonly used for monitoring the function of monocytes in clinical practice since it not only represents a costimulatory molecule but also a surrogate marker of monocyte anergy. A decreased expression of major histocompatibility complex (MHC) class II molecule has been well characterized for the monocytes isolated from septic patients. The levels of HLA-DR in non-survivors of septic shock were found to remain persistently low, and significant differences could be observed on days 3 to 4 after the onset of septic shock between the two groups, indicating its excellent capacity in the recognition and stratification of the septic patients (97). It was reported that the restoration of the mHLA-DR level could be observed at the follow-up for 6 months among discharged patients with sepsis. Moreover, a decreased mHLA-DR level was manifested to correlate with an increased risk of nosocomial infections after sepsis. Thus, dynamic monitoring of the level of mHLA-DR is capable of better assessing the immune status and predicting the prognosis of sepsis, further strengthened by its identical tendency with the CD4^+^ T cell counts (57, 97, 98). Correspondingly, it serves as a key marker of innate immune response in many interventional clinical trials as well as an essential indicator for monitoring the immune status during immunomodulation in septic patients (50, 51, 57, 79, 81).

Monocyte distribution width (MDW) represents a significant indicator reflecting monocyte response to bloodstream pathogenic invasions. The elevation of MDW has been documented for the early identification of sepsis (100–102). A high MDW level can potentially predict corticosteroid resistance among patients with sepsis and septic shock (103). A prospective cohort study indicated that upregulated monocyte PD-L1 expression was an independent risk factor of short-term mortality in septic shock (99). Since the proportion of CD14^+^HLA-DR^low^ monocytes was reportedly correlated with malignancy-related immunodeficiency, it largely contributed to the deteriorative survival rate, with unresponsiveness to immunotherapies (147). Although rare studies focused on the significance of CD14^+^ HLA-DR^low^ monocytes in human sepsis, latest advances using multi-omics methodologies implied that circulating CD14^+^HLA-DR^low^S100A^hi^ abundance was positively correlated with the severity of illness in patients with sepsis and severe acute respiratory syndrome coronavirus 2 infection (109, 110). Other than classical monocytes, a recently published study suggested that the proportion of CD14^-^CD16^+^ patrolling monocytes was negatively corelated with Sequential Organ Failure Assessment score in patients who developed post-traumatic sepsis, implicating a protective role of this subtype (111). Correspondingly, another study revealed that the absolute count of patrolling monocytes on day 3 higher than 27 cells/mm^3^ was negatively correlated with 28-day mortality among septic patients (33). Moreover, impairment in the phagocytic capacity of monocytes was expected to be a potential indicator in predicting persistent immunosuppression for septic patients (108).

The production of various pro-inflammatory cytokines is significantly reduced in monocytes isolated from septic patients with immunoparesis, including TNF-α, interleukin (IL)-1β, IL-6, and IL-12. Upon *in vitro* stimulation of LPS, circulating monocytes from septic patients showed a markedly diminished induction of TNF-α and IL-12 compared to that of the healthy individuals (104, 105). A threshold of 200 ng/L for *ex vivo* LPS-mediated TNF-α secretion was evident in recognizing the immunosuppressive state for septic patients (104). Meanwhile, a restored TNF-α level was deemed as a parameter regarding the responsiveness of immune-stimulatory treatment for septic patients. On the contrary, IL-10 and IL-1 receptor antagonists (IL-1Ra) are well established anti-inflammatory mediators associated with immunosuppression. The circulating monocytes were presented with an increased production of IL-10 in response to LPS stimulation, and they were closely associated with poor clinical outcomes of septic complications (105). A soluble peptide, namely, CD14 subtype (sCD14-ST) or presepsin, was demonstrated to have a close relationship with monocyte dysfunction, and its circulating level was applied as a candidate for early diagnosis and risk stratification in patients with sepsis and septic shock (106, 107). An observational study also highlighted the combined use of presepsin and mHLA-DR to show better performance in predicting a clinical prognosis of sepsis compared to that of single use (107). Strikingly, a group of septic shock patients were complicated with MAS, a clinical phenotype characterized by fever, hepatosplenomegaly, hepatobiliary dysfunction, and disseminated intravascular coagulation (148, 149). Those patients often have a rapidly progressing organ failure, with a significantly higher risk of early death (35). The pathogenesis involves overactivation of macrophages, for which a positive feedback loop of various proinflammatory mediators eventually leads to fulminant cytokine storm (36). Among them, elevated ferritin concentration has been well accepted as a diagnostic hallmark of MAS, which is also associated with unfavorable clinical outcomes in septic patients (150). Other than ferritin, the plasma levels of IL-1β, IL-6, IL-18, and sCD163 might serve as potential biomarkers in recognizing sepsis complicated with MAS (35, 149).

### Monitoring the Function and Proportion of Myeloid-Derived Suppressor Cells

It has been indicated that sepsis *per se* can result in the substantial augmentation of MDSCs in peripheral blood, representing one of the hallmarks of immunosuppressive response. Both circulating G-MDSCs and M-MDSCs were noted with significant increases at the early stage of sepsis, which were defined as CD14^-^CD15^+^ and CD14^+^CD15^-^ HLA-DR^–/low^, respectively (114). Nevertheless, G-MDSCs were reported to be more sensitive in discriminating septic and non-septic patients than M-MDSCs did (39). Moreover, G-MDSC frequencies were significantly correlated with long-term mortality in septic survivors and a high risk of septic shock (112, 114). Of note is that MDSCs acquired function after 2 weeks, while their proportions remained sustainably high for at least 6 weeks in patients with sepsis, in association with a long-term immunocompromised state and the onset of chronic critical illness (151, 152). Recently, the serum level of multiple mediators and the expression of various surface markers have been broadly applied to identify the emergence and immunosuppressive function of circulating MDSCs among septic patients, including S100A12, S100A8/A9, arginase 1 (ARG1), and lectin-type oxidized LDL receptor 1 (LOX-1) (112, 113).

### Monitoring the Function and Proportion of Dendritic Cells

A profound reduction of splenic DCs could be seen in patients who died of sepsis, and a depletion of circulating DC numbers was frequently reported in septic cases, which was critically involved in the development of septic shock and sepsis-induced immunosuppression (43, 45, 153). Importantly, reduction of DC counts could sustain for several weeks and was more evidently observed in non-survivors (45)—for example, Grimaldi et al. reported that both circulating plasmacytoid DC (CD123^+^HLA-DR^+^Lin^-^ cells) and myeloid DC (CD11c^-^HLA-DR^+^Lin^-^ cells) revealed a significant reduction in patients who developed septic shock (44). The expression of HLA-DR showed an obvious decrease on the surface of DCs from septic patients in comparison to that of the healthy controls, implicating a significantly impaired antigen-presenting function (115). This view was further strengthened by the subsequent study, in which enhanced IL-10 production was noticed in circulating DCs from patients with sepsis (49). Furthermore, the expression of B and T lymphocyte attenuator (BTLA), an immunoregulatory receptor on myeloid DCs, was found to positively correlate with the severity of illness among septic neonates, and BTLA^+^DCs constituted a tolerogenic and dysfunctional phenotype associated with an impaired capacity in potentiating T cell proliferation (117). Besides this, a high level of Blimp1, a transcription factor in driving the tolerogenic function of DCs, could be observed in circulating DCs isolated from septic patients, which was reportedly related to severity and clinical outcome (116). Therefore, assessing the number and function of DCs is essential for clinical practice by deeply understanding immunopathogenesis and exploration of an effective treatment (48).

### Monitoring the Function of Natural Killer Cells

The absolute counts and proportions of NK cells in lymphocytes were significantly decreased in septic patients, for which both CD56^hi^ and CD56^low^ NK cell subsets were consistently affected, in association with an increased risk of death (118, 121, 122). Conversely, higher proportions of NK cells indicated a better prognosis for septic patients (119). Similar to monocytes and neutrophils, an increased percentage of PD-L1^+^ NK cells was demonstrated to predict an increased risk of 28-day mortality in septic patients (123). NK cells exhibited a reduction in cytotoxicity in sepsis and septic shock patients, as evidenced by impaired killing capacity and the dampened expression of natural cytotoxicity receptors (NCRs) and NK group 2 member (NKG2) receptors, including NKG2C, NKG2D, NKp30, and NKp46 (118, 120). The measurement of these biomarkers on NK cells or NK cell subpopulations showed a good performance in the early diagnosis of sepsis, whereas their prognostic values remained largely divergent across studies (118, 120, 154). The immune-killing impact of NK cells is also achieved by releasing multiple cytokines, in which IFN-γ is the most representative one reflecting the function of NK cells. Identical to tolerant monocytes, the *ex vivo* release of IFN-γ by NK cells was greatly diminished upon LPS challenge, possibly in association with the reactivation of latent viruses in critically ill patients (56). Forel et al. found that the isolated NK cells from septic patients showed a significantly downregulated secretion of IFN-γ (119). A reduced production of TNF-α by NK cells was likely observed in patients with sepsis and septic shock (118). However, a contradictory conclusion was made by other investigators who reported a more powerful secretory capacity of NK cells in septic patients in comparison to that in healthy controls (155). The divergency might be attributed to the substantial patients’ heterogeneity as well as the inconsistent sampling intervals.

## Monitoring the Alterations of the Adaptive Immune System

Approximately 50% of septic patients are complicated with lymphopenia at diagnosis, and these patients show a higher risk than those with a normal count of lymphocytes in both mortality and developing chronic disorders (124, 156). Of note is that septic patients with a prolonged low count of lymphocytes (less than 1.1 × 10^9^/L) are more likely to develop immunosuppression, thereby leading to the higher risk of death (60, 124, 157).

### Monitoring the Function of T Lymphocytes

Sepsis-induced lymphopenia appears to contribute to the numerical loss of multiple subtypes of T cells, albeit at different degrees, given that the alteration in the T cell subtypes reveals great predictive value under a septic condition. By the use of flow cytometry, the decline in the ratio of CD4^+^/CD8^+^ T cells might serve as an indicator of abnormal adaptive immune response (131), which is significantly correlated with elevated Acute Physiology and Chronic health score II (APACHE II) scores and incidence of multiple organ dysfunction syndrome among patients with sepsis (132–134). Gupta reported that Th cells showed polarization toward Th2 subtypes under septic exposure, resulting in the imbalanced ratio of Th1/Th2, the extent of which could predict the prognosis of septic patients (135). However, another study came to a divergent result, and it showed a consistently attenuated differentiational capacity toward Th1, Th2, and Th17, as evidenced by diminished transcript levels of T-bet, GATA3, and ROR-γ T in patients with septic shock (105). It has been documented that Tregs (CD4^+^CD25^+^FOXP3^+^CD127^low/-^) play an indispensable role in potentiating long-term immunosuppression *via* promoting the apoptosis and suppressing the function of other Teff subsets, the expansion of which is commonly observed in the peripheral blood of septic patients (58, 72, 158, 159). The potential role of increased Tregs in the progression of sepsis remained controversial across studies: a report showed a close correlation of Treg proportion with poor prognosis (71), whereas others suggested the beneficial impact of enhanced Tregs on sepsis (137). Notably, the ratio of Th17/Treg usually presents with dynamic changes of early ascending and later descending during the course of sepsis. The abnormal elevation of Tregs is simultaneously followed with a reversed ratio of Th17/Treg, indicating the occurrence of an immunosuppressive state (138, 139). According to an early study, immunoparalysis was commonly seen in patients with septic shock, as shown by the increased representation of Tregs, which was especially noteworthy in non-survivors (136). Therefore, the relative count and balance between Th1, Th2, Th17, and Tregs are of prominent significance in the early identification of an immunocompromised state among patients with sepsis and septic shock. Grimaldi et al. conducted a study that evaluated the numerical alterations of circulating innate-like T lymphocytes and its correlation with clinical outcomes among critically ill patients with sepsis. They found that the depletion of CD3^+^TCRγδ^-^CD4^-^CD161^hi^Vα7.2^+^ MAIT cells rather than γδ T cells (CD3^+^TCRγδ^-^ cells) was associated with the increased incidence of ICU-acquired infections (74).

Emerging evidence implicates the pronounced functional defects of T cells among septic patients, and circulating T lymphocytes that survived from sepsis-induced apoptosis are presented with clonal anergy (29, 125). It has been reported that the proliferative activity of T cells shows a significant reduction in patients with severe trauma or burn injury and is critically involved in severity and high mortality, suggesting the hypoergia of T cells during the course of host immune dysregulation (126). Meanwhile, T cell exhaustion is characterized by upregulated expressions of various immune checkpoint molecules that dampen the immunopotency. Correspondingly, several studies revealed that circulating CD4^+^ T cells isolated from septic patients expressed a high level of inhibitory receptors, including programmed death-1 (PD-1), 2B4, and BTLA, which were associated with increased susceptibility to secondary infections and worsening clinical outcomes (62, 63, 127–129). Monitoring the secretory levels of cytokines represented one of the significant indicators for reflecting the function and differentiation of T cells, as the induction of multiple cytokines was greatly diminished in septic patients upon *ex vivo* stimulation, including IL-2, IL-6, IFN-γ, and TNF-α (34, 130).

### Monitoring the Proportion and Function of B Lymphocytes

In addition to the numerical loss, septic patients are presented with evident B cell dysfunction, as evidenced by the increased CD21^-/low^CD95^hi^ exhausted B cells in patients with sepsis and septic shock (80, 140, 141). This point was further strengthened by the decreased MHC-II expression and elevated IL-10 production of B cells that survived from sepsis-induced apoptosis, indicating an anergic profile (141). As for the alterations in subsets, reduced percentages of circulating plasmablasts and memory B cells could be observed in septic patients compared to that of the healthy controls, while the proportion of transitional B cells remained comparable (78). Reduction in CD19^+^CD27^+^ memory B cells and CD19^+^CD27^+^CD38^+^ plasmablasts, but not CD19^+^CD27^-^ naïve B cell population, was reported to predict 28-day mortality in septic settings (145). Moreover, the enhanced expressions of CD80 and CD95 on the surface of B lymphocytes were associated with a increased risk of death among septic patients, whereas CD23 expression was negatively correlated with an unfavorable outcome (144).

Regulatory B cells (Bregs) characterized by CD19^+^CD24^hi^CD38^hi^ cells exert a pronounced expansion in neonatal sepsis, and they appear to be critically involved in the development of host immune depression (146). It has been accepted that concentrations of serum IgG, IgA, and IgM usually serve as specific parameters to reflect the functional status of B cell directly. It was reported that the incidence of hypogammaglobulinemia due to IgG depletion reached 70% in septic patients but showed no specific connection to the clinical outcomes, which was further supported by the results of clinical trials (142, 143). Intriguingly, septic patients with a high serum level of IgG were more likely to die, implying that a high IgG level was a potential risk factor (143). Additionally, a decreased serum level of IgM in elderly septic patients was found to be related to the severity of illness (APACHE II score) and the occurrence of secondary infections after sepsis (80). Taken together, these results suggest that the value of a single Ig is far less than combining multiple kinds of Ig in predicting the prognosis of sepsis, and the combined use of serum IgG1, IgM, and IgA shows a good performance in predicting the clinical outcomes of septic patients (160).

## Conclusions and Prospects

Immune monitoring in sepsis is of prominent significance in identifying and stratifying septic patients with evident immunocompromised status and who are at a higher risk of recurrent infection and even long-term mortality (9). By interrogating the alterations in proportion and function of various immune cell subsets, clinicians can rapidly recognize patients with sepsis-induced immunosuppression, which subsequently guides and facilitates the implementation of adjunctive immunotherapies. The consecutive failure of multiple randomized controlled trials using immune stimulatory agents might be attributed to the substantial heterogeneity of septic patients and the lack of stratification based on their immune status (161–164). Correspondingly, potential biomarkers reflecting innate and adaptive immune functional states can, at least in part, eliminate heterogeneity and benefit septic patients from more personalized immune-adjuvant therapies.

**Figure 2:**
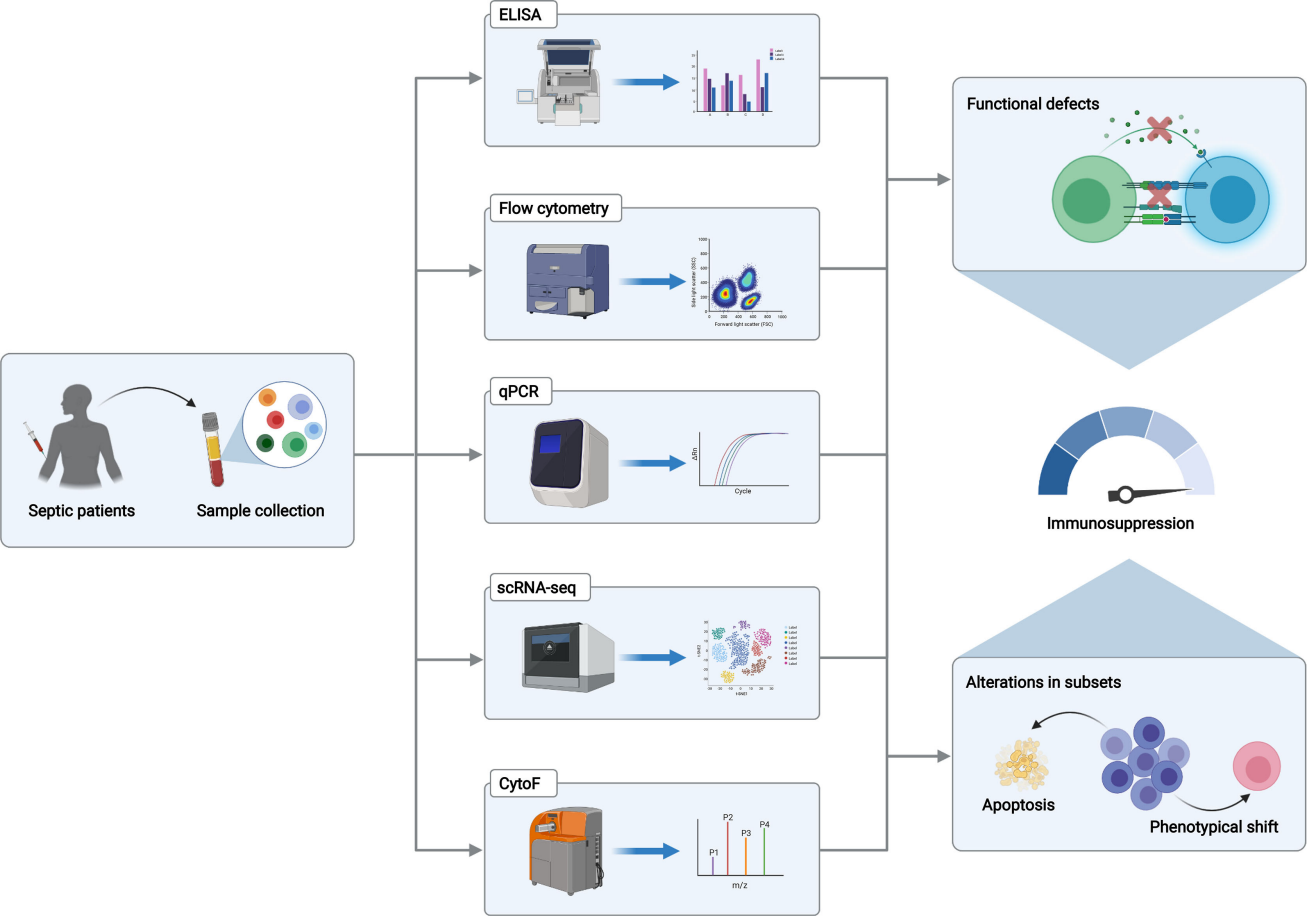
Approaches for immune monitoring of sepsis-associated immune dysfunction. Peripheral blood mononuclear cells or plasma isolated from septic patients are subjected to multiple immune monitoring assays in detecting the transcript and protein levels of various biomarkers that reflect functional status and subset alterations of certain immune cell types, including flow cytometry, ELISA, and qPCR. Moreover, transcriptomic- and proteomic-based sequencing technologies enable us to identify unique immune cell cluster and cell state, in association with sepsis-induced immunosuppression, including scRNA-seq and CytoF. The Graph was created with BioRender.com. ELISA, enzyme-linked immunosorbent assay; qPCR, quantitative real-time polymerase chain reaction; scRNA-seq, single-cell RNA sequencing; CytoF, cytometry with time-of-flight.

Although unprecedented progress has been made in the discovery of novel immune monitoring methods for septic patients, there are still major gaps hindering the application of these approaches (7). Firstly, one significant issue is that majority of immune-related biomarkers remain relatively unspecific, which are unable to convey the entire magnitude of immune dysregulation and to distinguish critically ill patients with or without infections. Leukocyte markers reflecting the changes in immune cell subpopulations can be potentially helpful (165). Meanwhile, the combined use of indicators like neutrophil-to-lymphocyte ratio and monocyte/high-density lipoprotein cholesterol ratio reportedly has a better performance in predicting mortality for septic patients than a single parameter did (166–168). Although numerous studies have documented that immune monitoring indicators can predict multiple clinical endpoints for septic patients, including short- or long-term mortality, hospital-acquired infections, and organ dysfunction, basically no specific biomarkers are found to achieve a consistently good performance in predicting all these outcomes (5). This divergency further highlights the necessity for the establishment of prediction models containing multiple immune-related parameters. Secondly, the lack of standardization of immunological measurement should be taken into consideration as well. Given the emerging applications of flow cytometry-based makers and transcriptome profiling methods, accurate detection of immune-relevant markers at the mRNA and protein levels is becoming a reality. Nevertheless, no authorized guideline or expert consensus is available in utilizing the thresholds of gene expression-based tests, hindering the generalization of these methods. Thirdly, the insufficient translation of many biomarkers and cutting-edge techniques represents an additional obstacle. Although several biomarkers are routinely used in clinical practice, including mHLA-DR, circulating IL-10, and CD4^+^/CD8^+^ ratio, the validity of many others is solely manifested in the pre-clinical studies using an experimental model of sepsis. In recent years, the development of multi-omics-based techniques enables researchers to decipher immune cell heterogeneity at single-cell resolutions, including mass cytometry and single-cell RNA sequencing (scRNA-seq), which have been broadly applied in various malignancies and autoimmune diseases (169–171). However, other than traditional techniques including flow cytometry and enzyme-linked immunosorbent assay (ELISA), merely a few studies adopt these methods in human sepsis, and they exert promising results in establishing novel gene sets associated with monocyte and neutrophil anergy (94, 109, 172) (**Figure 2**). Finally, the interplay between the microbiome and the immune system has been largely underscored since perturbations of intestinal microbiome can be constantly observed in septic patients, which plays an indispensable role in mediating post-sepsis immune dysregulation (173, 174). A study proposed a novel model in predicting lower respiratory tract infections among critically ill patients by integrating pathogen, airway microbiome, and host transcriptional profile, achieving a promising result (175). This study implicated that the combined use of parameters regarding microbiological components and host immune response might come out with an optimized performance in stratifying septic patients with distinct immune status. Meanwhile, the potential of microbiome-manipulating therapies in treating sepsis-induced immunosuppression requires further exploration. Given that, strengthening the translational medicine research and application of multi-omics methodologies can provide new insights into the molecular and cellular basis of sepsis-induced immune paralysis and facilitate the identification of novel yet feasible immune-relevant cell-type-specific disease signatures. Thus, it is our belief that the advancement of immune monitoring strategies can greatly prompt the prevention and treatment of sepsis.

## Author Contributions

Y-MY and Z-FX conceived the idea of this review. R-QY and CR performed literature search and co-wrote this paper. L-YZ conducted language editing and re-checking of literature. Y-MY checked and edited the content and format of this manuscript before submission. All authors contributed to the article and approved the submitted version.

## Funding

This work was supported by grants from the National Natural Science Foundation of China (numbers 81730057, 81801935, and 82130062), the Key Project of Military Medical Innovation Program of Chinese PLA (numbers 18CXZ026 and BLJ18J006), and CAMS Innovation Fund for Medical Sciences (2019-I2M-5-076).

## Conflict of Interest

The authors declare that the research was conducted in the absence of any commercial or financial relationships that could be construed as a potential conflict of interest.

## Publisher’s Note

All claims expressed in this article are solely those of the authors and do not necessarily represent those of their affiliated organizations, or those of the publisher, the editors and the reviewers. Any product that may be evaluated in this article, or claim that may be made by its manufacturer, is not guaranteed or endorsed by the publisher.
